# Effects of Budesonide Combined with Noninvasive Ventilation on PCT, sTREM-1, Chest Lung Compliance, Humoral Immune Function and Quality of Life in Patients with AECOPD Complicated with Type II Respiratory Failure

**DOI:** 10.1515/med-2019-0023

**Published:** 2019-03-02

**Authors:** Erxiang Gao, Chi Zhang, Jianping Wang

**Affiliations:** 1 Department of Emergency, 1st Hospital of Yulin, Yulin City, Shaanxi Province 719000, China; 2 Emergency Department, Xingyuan Hospital of Yulin, Shaanxi 719000, China; 3 The Second Department of Internal Medicine, Xingyuan Hospital of Yulin, Shaanxi 719000, China

**Keywords:** Budesonide, Noninvasive ventilation, Acute exacerbation of chronic obstructive pulmonary disease, Type II respiratory failure, Procalcitonin, Soluble myeloid cell triggering receptor-1, Thoracic and lung compliance, Humoral immune function, Quality of life

## Abstract

**Objective:**

Our objective is to explore the effects of budesonide combined with noninvasive ventilation on procalcitonin (PCT), soluble myeloid cell triggering receptor-1 (sTREM-1), thoracic and lung compliance, humoral immune function, and quality of life in patients with acute exacerbation of chronic obstructive pulmonary disease (AECOPD) complicated with type II respiratory failure.

**Methods:**

There were 82 patients with AECOPD complicated with type II respiratory failure admitted into our hospital between March, 2016-September, 2017. They were selected and randomly divided into observation group (n=41) and control group (n=41). The patients in the control group received noninvasive mechanical ventilation and the patients in the observation group received budesonide based on the control group. The treatment courses were both 10 days.

**Results:**

The total effective rate in the observation group (90.25%) was higher than the control group (65.85%) (P<0.05). The scores of cough, expectoration, and dyspnea were decreased after treatment (Observation group: t=18.7498, 23.2195, 26.0043, control group: t=19.9456, 11.6261, 14.2881, P<0.05); the scores of cough, expectoration, and dyspnea in the observation group were lower than the control group after treatment (t=11.6205, 17.4139, 11.6484, P<0.05). PaO2 was increased and PaCO2 was decreased in both groups after treatment (Observation group: t=24.1385, 20.7360, control group: t=11.6606, 9.2268, P<0.05); PaO2 was higher and PaCO2 was lower in the observation group than the control group after treatment (t=10.3209, 12.0115, P<0.05). Serum PCT and sTREM-1 in both groups were decreased after treatment (Observation group: t=16.2174, 12.6698, control group: t=7.2283, 6.1634, P<0.05); serum PCT and sTREM-1 in the observation group were lower than the control group after treatment (t=10.1017, 7.8227, P<0.05). The thoracic and lung compliance in both groups were increased after treatment (Observation group: t=30.5359, 17.8471, control group: t=21.2426, 13.0007, P<0.05); the thoracic and lung compliance in the observation group were higher than the control group after treatment (t=10.8079, 5.9464, P<0.05). IgA and IgG in both groups were increased after treatment (Observation group: t=9.5794, 25.3274, control group: t=5.5000, 4.7943, P<0.05), however IgM was not statistically different after treatment (Observation group: t=0.7845, control group: t=0.1767, P>0.05); IgA and IgG in the observation group were higher than the control group (t=4.9190, 4.7943, P<0.05), however IgM was not statistically different between two groups after treatment (t=0.6168, P>0.05). COPD assessment test (CAT) scores were decreased in both groups after treatment (Observation group: t=20.6781, control group: t=9.0235, P<0.05); CAT score in the observation group was lower than the control group after treatment (t=12.9515, P<0.05). Forced expiratory volume in one second (FEV1%) and forced expiratory volume in one second/ forced expiratory volume in one second (FEV1/FVC) were increased in both groups after treatment (Observation group: t=15.3684, 15.9404, control group: t=10.6640, 12.8979, P<0.05); FEV1% and FEV1/FVC in the observation group were higher than the control group (t=6.9528, 7.3527,P<0.05). The rates of complication were not statistically different between two groups (P>0.05).

**Conclusion:**

Budesonide combined with noninvasive mechanical ventilation has good curative effects in treating AECOPE patients complicated with type II respiratory failure. It can decrease serum PCT and sTREM-1, increase thoracic lung compliance, and improve the humoral immune function and life quality.

## 1 Introduction

Chronic obstructive pulmonary disease (COPD) is a common respiratory disease with an increasing incidence rate that has significantly affected the life quality of people [1, 2, 3]. Acute exacerbation of chronic obstructive pulmonary disease (AECOPD) can cause complications such as respiratory failure that endangers patients’ lives [4, 5, 6]. Thus, an effective curative method is especially important. Noninvasive positive pressure ventilation (noninvasive mechanical ventilation) is the primary method to treat AECOPD complicated with respiratory failure [5,7, 8, 9]. In the recent years, it is reported that aerosol inhalation of medicine can directly act on the airway, which in return has significant effect on AECOPD complicated with respiratory failure [9, 10, 11, 12]. Thus, in this study, we analyzed the effects of budesonide combined with noninvasive ventilation on procalcitonin (PCT), soluble myeloid cell triggering receptor-1 (sTREM-1), thoracic and lung compliance, and humoral immune function and quality of life in patients with AECOPD complicated with type II respiratory failure.

## 2 Materials and methods

### 2.1 General data

Eighty-two patients with AECOPD complicated with type II respiratory failure admitted into our hospital between March, 2016-September, 2017 were selected. The criteria was based on the diagnostic criteria of COPD [13]. The patients were divided into observation group (n=41) and control group (n=41). In the observation group, there were 15 female patients and 26 male patients. The age of patients was 50-78 years old and the average age was (65.98±4.87) years old; in the control group, there were 17 female patients and 24 male patients. The age of patients was 51-80 years old and the average age was (66.71±5.13) years old. The general data were comparable between two groups.

### 2.2 Inclusion and exclusion criteria

#### 2.2.1 Inclusion criteria

The patients met the criteria of AECOPD complicated with type II respiratory failure, and were also confirmed with past history, physical signs, blood routine test and X-Ray, and a severe AECOPD stage; the age of patients was 50-80 years.

Ethics approval and consent to participate: This study was approved by the Committee on Ethics of Biomedicine of Yulin First People’s Hospital. This study also complied with the Declaration of Helsinki, and signed, written informed consent was obtained from all subjects included in this study.

#### 2.2.2 Exclusion criteria

The patients complicated with severe electrolyte disturbance, acute cerebrovascular disease, or severe cerebral vessel sclerosis; the patients complicated with severe liver or kidney dysfunction; the patients had lots of secretion in the airway, which required emergent tracheal intubation; the patients had mental disorder.

### 2.3 Curative method

The patients in the two groups received routine treatment, including oxygen inhalation, bronchodilator, anti-infection and expectorant. Control group: noninvasive mechanical ventilation was applied as the following: a proper size nasal/face mask was selected and fixed with headband, the mask was connected to the ventilator, and then the ventilator parameters were set as the following: the inspiration pressure was initially 8-10 cmH_2_O and gradually increased to 12-20 cmH_2_O which fitted the tolerance of patients; the respiratory rate was 12-20/min; the oxygen flow rate was 4-6 L/min, the positive end-expiratory pressure was 3-5 cmH_2_O, most of patients received intermittent ventilation, and the duration was ≥4 h/time and ≥12 h/day. Observation group: the patients received series connection of budesonide suspension with ventilator circuit (AstraZeneca Pty Ltd., Sydney, Australia), 2 mg/time, 3 times/day. The treatment courses in both groups were 7 days.

### 2.4 Criteria for curative efficacy

Significant improvement: The major symptoms including cough, expectoration, and dyspnea almost disappeared, rale was significantly improved, chest imaging showed that the lung markings and shadow were significantly improved; improvement: The major symptoms including cough, expectoration and dyspnea were improved, rale was improved, chest imaging showed that the lung markings and shadow were improved; invalidity: The major symptoms including cough, expectoration, dyspnea, rale, and chest imaging were not improved. The total effective rate=(case number of significant improvement+case number of improvement)/total case number×100%.

### 2.5 Observational indexes

The major symptoms (cough, expectoration, and dyspnea) before and after treatment in two groups were observed and evaluated as none (0 point), mild (1 point), moderate (2 points), and severe (3 points) based on the score; the changes of blood gas parameters before and after treatment in the two groups were observed; the changes of PCT and sTREM-1 before and after treatment in the two groups were observed: first 3 ml peripheral venous blood was collected before and after treatment and the serum was separated by centrifugation at 15 cm diameter and 2500 r/min for 24 hours; the changes of thoracic and lung compliance before and after treatment in the two groups were observed; the changes of humoral immune function before and after treatment in the two groups were observed, including IgA, IgG and IgM in above serum sample detected by Hitachi 7600 modular chemistry analyzer (Hitachi, Ltd., Tokyo, Japan); the improvement of life quality before and after treatment in the two groups were observed, COPD assessment test (CAT) was applied including 6 subjective indexes of tight chest, cough, energy, phlegm, emotion and sleep and 2 tolerance indexes including exercise tolerance and daily exercise influence, 5 points per item, and the total CAT scores range from 0-40. Higher scores denote a more severe impact of COPD on a patient’s life; pulmonary functions in both groups before and after treatment were observed, including forced expiratory volume in one second (FEV1), forced vital capacity (FVC) and a percentage of the forced vital capacity (FEV1%); the complications in two groups were observed.

### 2.6 Statistical analysis

The enumeration data and measurement data were analyzed by SPSS13.0. All the statistical significance was presented as *P<0.05* The enumeration data were analyzed by *x^2^* and presented as percentage; the measurement data were analyzed by *t* test and presented as mean±SD.

## 3 Results

### 3.1 Comparison of total effective rate between two groups

As shown in Table 1, the total effective rate in the observation group (90.25%) was higher than control group (65.85%) (*P<0.05*).

**Table 1:** Comparison of total effective rate between two groups

Group	Case number	Significant improvement (%)	Improvement (%)	Invalidity (%)	Total effective rate (%)
Observation group	41	23(56.10)	14(34.15)	4(9.75)	37(90.25)
Control group	41	14(34.15)	13(31.70)	14(34.15)	27(65.85)
x2	-	-	-	-	7.1181
P	-	-	-	-	<0.05

### 3.2 Comparison of major symptoms between two groups

As shown in Table 2, the scores of cough, expectoration, and dyspnea were not statistically different between two groups before treatment; the score of cough, expectoration, and dyspnea were decreased after treatment (Observation group: *t*=18.7498, 23.2195, 26.0043, control group: *t*=19.9456, 11.6261, 14.2881, *P<0.05*); the scores of cough, expectoration, and dyspnea in the observation group were lower than the control group after treatment (*t*=11.6205, 17.4139, 11.6484, *P<0.05*).

**Table 2:** Comparison of major symptoms between two groups (*x̄x̄*±s)

Group		Case number	Cough (point)	Expectoration (point)	Dyspnea (point)
Observation group	Before treatment	41	1.94±0.45	1.87±0.39	1.82±0.31
	After treatment	41	0.56±0.14ab	0.41±0.10ab	0.47±0.12ab
Control group	Before treatment	41	1.96±0.40	1.85±0.45	1.84±0.36
	After treatment	41	1.08±0.25a	0.97±0.18a	0.91±0.21a

Note: ^a^*P<0.05* compared with before treatment in the same group; ^b^*P<0.05* compared with the control group after treatment.

### 3.3 Comparison of blood gas parameters between two groups before and after treatment

As shown in Table 3, PaO_2_ and PaCO_2_ were not statistically different between two groups before treatment (*t*=1.1743, 0.4931, *P>0.05*); PaO_2_ was increased and PaCO_2_ was decreased in both groups after treatment (Observation group: *t*=24.1385, 20.7360, control group: *t*=11.6606, 9.2268, *P<0.05*); PaO_2_ was higher and PaCO_2_ was lower in the observation group than the control group after treatment (*t*=10.3209, 12.0115, *P<0.05*).

**Table 3:** Comparison of blood gas parameters between two groups before and after treatment ( *x̄x̄*±s)

Group		Case number	PaO_2_ (mmHg)	PaCO_2_ (mmHg)
Observation group	Before treatment	41	52.18±2.45	65.98±3.25
	After treatment	41	70.38±4.16^ab^	52.13±2.78^ab^
Control group	Before treatment	41	52.85±2.71	66.34±3.36
	After treatment	41	61.32±3.78^a^	59.83±3.02^a^

Note: ^a^*P<0.05* compared with before treatment in the same group; ^b^*P<0.05* compared with the control group after treatment.

### 3.4 Comparison of serum PCT and sTREM-1 between two groups before and after treatment

As shown in Table 4, serum PCT and sTREM-1 were not statistically different between two groups before treatment (*t*=0.4159,0.6173,*P>0.05*); Serum PCT and sTREM-1 in both groups were decreased after treatment (observation group: *t*=16.2174, 12.6698, control group: *t*=7.2283, 6.1634, *P<0.05*); serum PCT and sTREM-1 in the observation group were lower than the control group after treatment (*t*=10.1017, 7.8227, *P<0.05*).

**Table 4:** Comparison of serum PCT and sTREM-1 between two groups before and after treatment ( *x̄x̄*±s)

Group		Case number	PCT (ng/L)	sTREM-1(ng/L)
Observation group	Before treatment	41	613.25±24.25	94.52±3.67
	After treatment	41	536.29±18.31^ab^	85.41±2.78^ab^
Control group	Before treatment	41	615.46±23.87	95.03±3.81
	After treatment	41	579.83±20.65^a^	90.38±2.97^a^

Note: ^a^*P<0.05* compared with before treatment in the same group; ^b^*P<0.05* compared with the control group after treatment.

### 3.5 Comparison of humoral immune function between two groups

As shown in Table 5, IgA, IgG and IgM were not statistically different between the two groups before treatment (*t*=0.5486, 0.1484, 0.3412, *P>0.05*); IgA and IgG in both groups were increased after treatment (Observation group: *t*=9.5794, 25.3274, control group: *t*=5.5000, 4.7943, *P<0.05*), however IgM was not statistically different in both groups after treatment (observation group: *t*=0.7845, control group: *t*=0.1767, *P>0.05*); IgA and IgG in the observation group were higher than the control group (*t*=4.9190, 4.7943, *P<0.05*), however IgM was not statistically different between two groups after treatment (*t*=0.6168, *P>0.05*).

**Table 5:** Comparison of humoral immune function between two groups ( *x̄x̄*±s)

Group		Case number	IgA (g/L)	IgG (g/L)	IgM (g/L)
Observation group	Before treatment	41	1.94±0.17	10.12±1.21	1.36±0.25
	After treatment	41	2.38±0.24^ab^	12.89±1.37^ab^	1.40±0.21
Control group	Before treatment	41	1.92±0.16	10.08±1.23	1.38±0.28
	After treatment	41	2.14±0.20^a^	11.42±1.30^a^	1.37±0.23

Note: ^a^*P<0.05* compared with before treatment in the same group; ^b^*P<0.05* compared with the control group after treatment.

### 3.6 Comparison of thoracic and lung compliance between two groups before and after treatment

As shown in Table 6, thoracic and lung compliance were not statistically different between the two groups before treatment (*t*=0.5551, 0.5778, *P>0.05*); The thoracic and lung compliance in both groups were increased after treatment. (Observation group: *t*=30.5359, 17.8471, control group: *t*=21.2426, 13.0007, *P<0.05*); The thoracic and lung compliance in the observation group were higher than the control group after treatment (*t*=10.8079, 5.9464, *P<0.05*).

**Table 6:** Comparison of thoracic and lung compliance between two groups before and after treatment ( *x̄x̄*±s)

Group		Case number	Thoracic compliance	Lung compliance
Observation group	Before treatment	41	583.25±27.41	320.93±17.42
	After treatment	41	793.42±34.51^ab^	394.52±19.84^ab^
Control group	Before treatment	41	579.94±26.58	318.76±16.58
	After treatment	41	715.21±30.92^a^	369.29±18.56^a^

Note: aP<0.05 compared with before treatment in the same group; bP<0.05 compared with the control group after treatment.

### 3.7 Comparison of CAT score between two groups before and after treatment

As shown in Table 7, CAT scores were not statistically different between the two groups before treatment (*t*=0.3993,*P>0.05*); CAT scores were decreased in both groups after treatment (observation group: *t*=20.6781, control group: *t*=9.0235, *P<0.05*); CAT score in the observation group was lower than the control group after treatment (*t*=12.9515, *P<0.05*).

**Table 7:** Comparison of CAT score between two groups before and after treatment ( *x̄x̄*±s)

Group		Case number	CAT(point)
Observation group	Before treatment	41	28.42±3.15
	After treatment	41	16.59±1.87ab
Control group	Before treatment	41	28.15±2.97
	After treatment	41	22.76±2.41a

Note: ^a^*P<0.05* compared with before treatment in the same group; ^b^*P<0.05* compared with the control group after treatment.

### 3.8 Comparison of pulmonary functions between two groups before and after treatment

As shown in Table 8, FEV1% and FEV1/FVC were not statistically different between the two groups before treatment (*t*=0.8258, 1.2307, *P>0.05*): FEV1% and FEV1/FVC were increased in both groups after treatment (Observation group: *t*=15.3684, 15.9404, control group: *t*=10.6640, 12.8979, *P<0.05*); FEV1% and FEV1/FVC in the observation group were higher than the control group (*t*=6.9528, 7.3527, *P<0.05*).

**Table 8:** Comparison of pulmonary functions between two groups before and after treatment ( *x̄x̄*±s)

Group		Case number	FEV1% (%)	FEV1/FVC (%)
Observation group	Before treatment	41	51.32±4.97	54.28±3.91
	After treatment	41	68.97±5.42^ab^	70.16±5.04^ab^
Control group	Before treatment	41	50.28±6.35	53.17±4.25
	After treatment	41	62.13±3.21^a^	63.50±2.87^a^

Note: ^a^*P<0.05* compared with before treatment in the same group; ^b^*P<0.05* compared with the control group after treatment.

### 3.9 Comparison of complications between two groups

Among 41 patients in the observation group, there was hyperglycaemia in one patient; among 41 patients in the control group, there were hyperglycaemia in 3 patients and upper gastrointestinal hemorrhage in one patient. The rates of complications were not statistically different between the two groups (*P>0.05*).

## 4 Discussion

COPD mainly involves lungs and can also cause extrapulmonary manifestations such as malnutrition, anxiety, depression, and osteoporosis [14, 15, 16]. As a development of environmental pollution and population aging, the incidence rate, disability rate, and mortality rate of COPD are increased [17, 18, 19]. The repetitive acute exacerbation of COPD can cause continuous decrease of lung function mostly complicated with malnutrition, which causes poor immune function and respiratory failure [6,20,21].

In the recent years, it has been proven that noninvasive mechanical ventilation has a good effect on patients with COPD complicated with respiratory failure. It is convenient, efficient, and good for observing the patient condition and supportive treatment, which significantly decreases the tracheal intubation rate, mortality, and length of stay in the hospital [22, 23, 24]. In noninvasive positive mechanical ventilation, the positive end-expiratory pressure and pressure support ventilation are combined to give pressure support during inspiration to decrease the load of respirator muscle and overcome the airway resistance; it is helpful to open the airway, promote the even distribution of air in the lung and oxygen diffusion, and avoid alveolar collapse. Furthermore, it can improve the air distribution and ventilation/perfusion ratio, which is helpful to the expiration of CO_2_ [25, 26, 27]. Aerosol inhalation of glucocorticoid is the main method of treating COPD complicated with respiratory failure at present, and budesonide is one of the preferred medicines [28,29]. Budesonide is a novel synthetic non-halogenate hormone with high lipophicity. Aerosol inhalation of budesonide can directly function on the disease site and rapidly improve the symptoms of COPD. It is reported that aerosol inhalation of budesonide can decrease airway resistance and pressure, effectively improve blood gas parameters, and shorten duration of mechanical ventilation and length of stay in hospital [30,31]. The results in this study showed that the total effective rate in the observation group was higher than the control group, IgA and IgG in the observation group were higher than the control group after treatment, CAT score in the observation group was lower than the control group after treatment, and FEV1% and FEV1/FVC in the observation group were higher than the control group. This suggests that budesonide combined with noninvasive mechanical ventilation can improve the curative effect, improve the symptoms, improve the thoracic and lung compliance of patients, improve the humoral immune function of patients, and improve the life quality of patients. The results in this study showed that serum PCT and sTREM-1 in the observation group were lower than the control group, suggesting that budesonide combined with noninvasive ventilation can decrease serum PCT and sTREM-1 level. PCT is a glycoprotein and the propeptide of calcitonin. PCT concentration is significantly increased in the early stage even when there are atypical clinical manifestations or immune suppression, and the increase degree is positively related to the infection severity [32,33]. sTREM-1 is a member of the immune globulin superfamily related to inflammation, which is mainly expressed in myeloid cells such as macrophage, monocyte, and neutrophils. When there is infection with extracellular bacteria, sTREM-1 expression is significantly up-regulated [34, 35, 36_]_.

In conclusion, Budesonide combined with noninvasive mechanical ventilation has good curative effects in treating AECOPE patients complicated with type II respiratory failure. It can decrease serum PCT and sTREM-1, increase thoracic lung compliance, and improve the humoral immune function and life quality.
